# A Dialogue about Vaccine Side Effects: Understanding Difficult Pandemic Experiences

**DOI:** 10.1007/s10912-024-09850-4

**Published:** 2024-07-01

**Authors:** Mia-Marie Hammarlin, Pia Dellson

**Affiliations:** 1 Department of Communication and Media, Lund University, Lund, Sweden; 2 Birgit Rausing Centre for Medical Humanities, Lund University, Lund, Sweden; 3 Department of Clinical Sciences Lund, Lund University, Skane University Hospital, Lund, Sweden

**Keywords:** vaccine hesitancy, side effects, COVID-19, swine flu, narcolepsy, dialogue

## Abstract

This paper investigates the relationship between the experiences of mass vaccinations against two pandemic viruses: the swine flu in 2009–2010 and COVID-19 in the early 2020s. We show how distressing memories from the swine flu vaccination, which led to the rare but severe adverse effect of narcolepsy in approximately 500 children in Sweden, were triggered by the COVID-19 pandemic. The narcolepsy illness story has rarely been told in academic contexts; therefore, we will provide space for this story. It is presented through a dialogue with the aim of shedding light on the interrelationship between pandemics—and between mass vaccinations—to investigate what could be termed cultural wounds that influence societies because they are characterized by the difficulty of talking about them. The paper explores the multiple shocks of illness in life and what can be learned from them by sharing them.

## Introduction

At times, measures against pandemics have unforeseen consequences. In 2009, the A(H1N1) influenza (a.k.a. the swine flu) spread rapidly around the world. In an effort to control the pandemic, new vaccines were developed and implemented, among them Pandemrix, produced by the pharmaceutical industry company GlaxoSmithKline (GSK). Subsequently, it was discovered that a number of individuals, especially children and young adults, who had received the Pandemrix vaccine developed narcolepsy as a side effect.

Narcolepsy is a severe neurological chronic disease characterized by that the brain cannot control wakefulness and sleep. Narcolepsy brings great suffering to affected patients, strongly impacting also on their relatives. The disease is characterized by excessive daytime sleepiness, sudden sleep attacks, paralysis, hallucinations while falling asleep or awakening, and cataplexy, which is a sudden loss of muscle tone and control, usually triggered by strong emotions such as laughter or excitement. This can cause the person to collapse or temporarily become weak and unable to move. Narcolepsy as a Pandemrix adverse effect was observed in Sweden, Norway, and Finland, but it also occurred elsewhere in northern Europe and has led to many investigations and publications. In 2011, the European Medicines Agency recommended restricting the use of Pandemrix (European Medicines Agency 2011).^1^

The aim of this paper is to investigate the adverse effects of the Pandemrix vaccine beyond but also including the strictly medical domain. We do so through a dialogue between two distinct academic and personal perspectives, shedding light on, firstly, narcolepsy as an illness experience and, secondly, the interrelatedness between the swine flu mass vaccination in 2009–2010 and the COVID-19 mass vaccination in 2021, from a Swedish horizon. It is we, the authors of this study, who engage in this dialogue with each other. Following in the steps of MacInnis and Portelli (2002), it is a meeting between two specialists from two different disciplines: Pia Dellson is an oncologist and psychiatrist, and Mia-Marie Hammarlin is an ethnologist and media scholar. We are both knowledgeable in relation to the subject at hand, Mia-Marie as the primary investigator on a research project^2^ on vaccine hesitancy (see Hammarlin 2022, 2023), and Pia as a medical poet and “mom-doctor” to Axel,^3^ her youngest son who developed narcolepsy after the swine flu vaccination (Dellson 2015). We are both fellows of the Birgit Rausing Centre for Medical Humanities at Lund University. In this paper, we at times refer to ourselves as “us” or “we” and, at times, as “Pia” or “Mia-Marie.”

Since we started to work together at the Birgit Rausing Centre, we have had numerous spontaneous dialogues about vaccine hesitancy and side effects. Most often, they have taken place in Pia’s living room with only the two of us present. But on one occasion, Axel strolled down the stairs at noon in his robe with tangled hair and a friendly smile on his face, not as a symbol of narcolepsy but as a reminder of stories that say, “… *these things happen; I embody what happened*” (Frank 2012, 76; italics in original). Mia-Marie smiled back and said, “My teenage daughter also sleeps until noon as soon as she gets the chance!” The morning after, Mia-Marie wrote a text message to Pia: “The first thing I did was comment upon Axel’s sleep. I feel so stupid! I was just looking for a subject that could suit a teenager.” Pia answered, “No worries, sleeping issues are, for both Axel and us as parents, the most natural thing in the world to talk about—we do it all the time.” This initial anxiety on Mia-Marie’s behalf says something important about the ramifications of severe illness; it becomes an issue to deal with, where avoidance is always a potential social and cultural response, at least in Sweden.

After some time, we took our conversation to the workshop “Medical Humanities and COVID/Post-COVID challenges,” organized by Linköping University’s Centre for Medical Humanities and Bioethics in September 2022. There, to a small international academic audience, we performed a dialogue that, due to the time frame, was simultaneously structured and spontaneous. Three months later, we returned to the subject and recorded and transcribed a dialogue between us, which formed the material on which this paper is based. But before accounting for our methodological and theoretical choices, we need to remind the reader of the swine flu in some more detail.

## The Tragic Consequences of the Pandemrix Vaccination: A Short Background

The A(H1N1) influenza pandemic spread rapidly around the world in 2009. The Swedish state chose to recommend vaccination of the entire population. Around 1.5 million Swedish children were vaccinated. Since then, 740 reports of narcolepsy have been made, and for about 440 of them, the state has assessed that the disease was caused by the Pandemrix vaccine.^4^ In the end, the swine flu turned out to be rather mild. According to the Swedish authorities, approximately 60 lives were saved due to mass vaccination.^5^

Pia, Axel, and the rest of the family were vaccinated with Pandemrix in 2009, and at the age of six, Axel developed narcolepsy as a side effect. It took some time before Pia and her husband realized that he had developed a severe medical condition and sought medical care. Axel was eight years old when he was diagnosed and has since been put on symptom-relieving narcotic-classified medication. He takes three doses of medicine at night to obtain deep sleep and two doses during the day to be able to stay awake as much as possible. He also naps twice during the day. From the Swedish state, he has received compensation of 5,000 euros for pain and suffering and 7,000 euros for medical disability. He may receive more compensation in the future if he can prove that the narcolepsy has affected his education and livelihood (we will return to this question below). After thorough discussions and research, the entire Dellson family chose to vaccinate themselves against COVID-19.

In a study of online conversations among Swedish vaccine-hesitant citizens taking place in 2020–2021 concerning the possibility of avoiding the COVID-19 vaccination, Mia-Marie and her colleagues found that the fear of side effects was strong, a common argument in general when being hesitant towards vaccines (WHO 2014). The most common side effect mentioned in the analyzed material was narcolepsy, despite it not having any connection to the COVID-19 vaccines being discussed (Hammarlin, Kokkinakis, and Borin 2023). It seems, instead, that a *collective memory formation* (Hammarlin, Kokkinakis, and Borin 2023, 53, italics in original) had taken place in the discussion threads, that had been ongoing since the swine flu in 2009. This indicates that, in peoples’ minds, one pandemic is related to another and, thus, that mass vaccinations are not perceived separately by people but in relation to one another.

Since the swine flu, narcolepsy-affected children have become quite well-known in Sweden. Many people relate to them in one way or another; either people know someone who has been affected or they know someone who knows someone. The media has also helped people to recall what happened.^6^ In many ways, the fact that children developed narcolepsy because of a vaccine turned them into a concern for everyone. This is something that Pia has experienced on several occasions when people come up to her and ask considerate questions about Axel. But concerned attention also has an opposite pole—silence. In this paper, we address and challenge the disengaged silence about the adverse vaccine effects of Pandemrix, especially on the part of public agencies, by engaging and talking. A first step in resisting silence is to make lives possible to narrate, thereby making them morally recognizable (Frank 2012, 75). This is especially important regarding vaccine-caused side effects, as “People prefer to keep nonnarratable what they want to believe did not or does not happen” (Frank 2012, 76; c.f. Mezza and Blume 2021). We wish to highlight the cultural and social aspects of vaccine adverse effects by asking the following questions: What does a severe medical condition like narcolepsy do to someone’s life? And how do intimate experiences of negative events following vaccination affect people’s willingness to get future vaccines?^7^ And lastly, how can a tragedy like narcolepsy as a vaccine side effect be collectively processed and remembered and, thus, healed?

In February 2023, 479 people who had become sick with narcolepsy following the Pandemrix vaccination made a collective claim for damages directed towards the Swedish state for compensation of 363 million Swedish krona (32 million euros).^8^ It was based on the argument that the Swedish state violated Article 8 of the European Convention on Human Rights, which says that nations must actively provide people with the necessary information so that they can assess risks to life and health and that they should only implement pressure for vaccination if there is a strong societal need. A decision in favor of the families would, they claimed, finally confirm the state’s responsibility for the vaccination injuries. In June 2023, the claim was dismissed (Justititiekanslern Dnr 2023). The Chancellor of Justice concluded that there had been no obligation for the state to inform the population about the, at the time, unknown increased risk of developing narcolepsy as a consequence of the vaccination. Nor had the state any obligation to specifically inform about the testing procedure established within the European Union that preceded the approval of the vaccine, and it had therefore not violated any rights.

However, lessons have been learned. As a direct consequence of the swine flu experiences, the Swedish state established a national vaccination and health data register, regulated by law, which is aligned with other Nordic countries’ national vaccination registers. This replaced the earlier regional register in Sweden to, among other things, facilitate side effects surveillance.^9^

In relation to the events following Pandemrix, the medical ethicist Nihlén Fahlquist (2018) writes, and we quote her at length:

The concerns of lay people should not be seen as signs of ignorance, but as a starting-point for a responsible and respectful discussion. Furthermore, the government should take full responsibility for individuals affected by side effects, i.e. for the cases where people are harmed instead of helped by policies and health-promoting measures recommended by the government. As a society, we probably have to accept some side effects, e.g. individuals affected negatively by public health policies, but this makes it crucial to see to it that individuals who are harmed instead of helped are taken care of. This is important for normative ethical reasons, and also in order to restore and maintain trust in experts and vaccines. (Nihlén Fahlquist 2018, 187)

The fear of vaccine side effects, we argue, should be met by empathy to avoid stigmatizing those who are hesitant towards inoculation (Larson and Broniatowski 2021; Drążkiewicz 2022; Hammarlin 2022; Hammarlin, Kokkinakis, and Borin 2023; Hausman 2019; Lundgren 2015a; Lundgren 2015b). They are not necessarily “anti-vaxx,” although they may consume content from anti-vaccination organizations when searching for facts that confirm or dispel their worries (Larson and Broniatowski 2021). Finding themselves somewhere in the middle of a continuum ranging from total acceptance to complete refusal (Larson and Broniatowski 2021; Dubé and MacDonald 2022; WHO 2014, 8), they are vulnerable to manipulation and risk being judged by others and also by health care professionals who are positioned to encourage vaccinations. The vaccine hesitant are a highly heterogeneous group, and there may be personal experiences of illness and suffering that make them indecisive towards inoculation, stories that should not be dismissed but listened to (à Rogvi and Hoeyer 2024, 89–108).

Similar to the majority of the Swedish population, we, the authors of this article, have a strong scientifically based belief in vaccinations, a medical intervention that, over time, has saved billions of lives globally, COVID-19 vaccines just being one example among many. The cases of narcolepsy caused by Pandemrix demonstrate, however, that severe vaccine side effects could still occur despite Sweden’s highly efficient pandemic preparedness. Ironically, it seems that they occurred *because of* a well-planned purchase agreement with vaccine producers and successful implementation, Lundgren (2015a, 162) argues.

In line with both Lundgren (2015a) and Nihlén Fahlquist (2018), we believe that when things go wrong, they should be transparently addressed and publicly discussed.

## Material

The dialogue on which this paper builds is an exchange between two people who consider themselves to be equals (McInnis and Portelli 2002, 34); we have a mutual respect for one another’s knowledge on vaccination hesitancy. The dialogue was recorded and later transcribed verbatim by our assistant, Jullietta Stoencheva, who also helped us translate it into English. Similar to our conference presentation mentioned earlier, we referred to Pia’s poetry during the dialogue. Some of the poems are published in the book *Sovsjuk: En mammadoktor skriver om narkolepsi* (2015) [Sleeping Sick: A Mom Doctor Writes about Narcolepsy], while others are yet unpublished. Pia is responsible for the translation of her poetry from Swedish to English. As usual, the dialogue took place in Pia’s living room and lasted for nearly two hours. We do not talk as well structured as we write, so the language has been slightly polished to reduce repetitions, interruptions, and dead-ends, and it has also been shortened to fit the article format. After several readings, recurrent themes were identified that sometimes have been put together, which is marked in the text by square brackets.

This process points to the issue of interpretation. Interpreting our own dialogue in detail is impossible, at least for us. Concurrently, it is as futile to try to avoid interpretation as it takes place already when reading and editing the transcript. We, therefore, rely upon the presumption that the openness of dialogue should mirror the openness of interpretation. In this particular case, we strive for “letting stories breathe” (Frank 2012), recognizing that people—not only researchers—are constantly doing their own narrative analyses and making sense of the stories they hear (Frank 2012, 16). So, to a certain extent, we leave the interpretation to the reader. Nevertheless, the article ends with a critical discussion.

To become a research dialogue, the conversation should contain subjective experiences and perspectives (MacInnis and Portelli 2002, 35). At the same time, this subjectiveness runs the risk of being transformed into “facts” and “truths.” We are cautiously aware of this risk and strive to transparently inspect the claims that are being made in our conversation. This, in turn, points to the elusiveness of memory. Portelli (1997) recognizes how difficult it can be for people to tell the truth when accounting for their struggles and painful search for meaning (see, also, Frank 2012, 91), with their stories mixing memories with narrative conventions and expectations about the listeners’ needs. However, no memory is free of distortion. Even the very idea of distortion could be seen as problematic in relation to memory, suggesting that there would exist such a thing as transparent recall, where distortion is seen as an exception (Frank 2012, 91). In fact, the reconstitution of memory is so common that calling it a distortion misrepresents what memories are and also what the truth of stories is (Frank 2012, 91). This reasoning leads us to a deeper exploration of dialogue.

## Dialogue: Methodological Perspectives

What do we mean by dialogue? On the one hand, as explained above, it is what the reader may expect it to be: namely, a conversation between two fellow humans. On the other hand, dialogue forms the epistemological starting point for this investigation. Building on Mikhail Bakhtin’s (1895–1975) philosophy of language, in particular his concept of *dialogism*, and his renowned interpreters, such as Michael Holquist (2002), we regard dialogue as something much more complex than the actual speech situation.

Dialogue points to the relationship between selves and others; in fact, the subject, the “self,” is dialogic to begin with. For Bakhtin, the self can never be a self-sufficient construct. Rather, it is a *relation*, expressed as self/other (Holquist 2022, 19). And whatever else it may be, the relation self/other is a relation of simultaneity that happens in the active flow of everyday life in the form of, among other things, primary and simple speech (Bakhtin 1986, 61), what Frank perhaps would recognize as the movement of thought in dialogue (Frank 2012, 73). This points to Bakhtin’s view of *being* as an event, something that happens, calling it “the unique and unified event of being” (Holquist 2002, 24). And the event of being is a matter of *co-being*; it is always shared (Holquist 2002, 24–25). In fact, for Bakhtin, there is no choice but to enter into dialogue as the self has its ongoing existence and its origin only in that realm; living is participating in dialogue (Frank 2005, 45; Bakhtin [1984] 2003, 252).

Still, the self does experience unique things in life. For example, there is a space that only “I” occupy in the world. If I happen to hit my head hard and develop a concussion as a consequence, the pain and confusion happen to *me*; it takes place in my individual body (Holquist 2002, 24). The Other can intellectually and emotionally empathize with me, but they cannot feel my pain in the same way that I do. But however unique the experience of being may come across, the event of existence is unified, “for although it occurs in sites that are unique, those sites are never complete in themselves” (Holquist 2002, 24–5).

Over time, the American sociologist Arthur Frank has made an impressive contribution to the field of illness narrative analysis. In some of his works (Frank 2005, 2012), he makes an effort to explore experiences of illness through Bakhtin’s dialogue theories. We will draw on Frank to delimit our contribution and, hopefully, make it more useful. Our dialogue is not about just any event; it explores the multiple shocks of illness in life (Frank 2013, 6) and what can be learned from them by sharing them. Only a tiny body of scholarly publications regarding Pandemrix-caused narcolepsy shed light on the affected, their families, and their stories of suffering (Lundgren 2015, 2015b, 2016, 2017).^10^ We see this lack as a sign of the “edges of a wound that can only be told around” (Frank 2013, 98). What we create through our dialogue is a *moral moment*: an occasion when one cannot but respond to another person. The nature of that response says something important about one’s moral self (Frank 2005,19). As the moral moment is revealing, trying to avoid it may be an instinct, Frank writes and continues, “But then the mirror shows us seeking to escape—the moral moment cannot be evaded” (Frank 2005,19). In this context, through sharing our dialogue, we would like to invite the reader to respond in the metaphorical sense of the word. The necessity of response is formulated by Lundgren: “As long as it is not ignored, this side effect can enable the possibility to create strengthened reflexive awareness, which in turn strengthens public trust regarding possible future [pandemic] interventions” (Lundgren 2016, 1108).

So, the task that we have given ourselves is to share with you, the reader, individual experiences that point to our common existence, providing space for the relation between the self and the Other. As already mentioned, unlike most scholarly publications, the researchers’ personal experiences are not excluded in our article but are seen as an important part of the dialogical process (McInnis and Portelli 2002, 35). As a matter of fact, it is the personal experiences of narcolepsy that Pia has as Axel’s mother that formed the very starting point of this inquiry. Pia is to be seen as an expert on the troubles that she has experienced in everyday life for many years due to her son’s illness. And there is, we claim, some degree of generalizability in relation to the other affected families in Sweden who continuously struggle for recognition; while Pia’s story is founded in personal memories, thoughts, and feelings, it also encompasses her membership in groups such as the Association for Narcolepsy.^11^ She does not act as a representative for the association in the dialogue, but we do believe that individual problems, in general, are ultimately community problems (Frank and Nyheim Solbraekke 2023, 81). Mia-Marie is the active listener in the dialogue and participates mostly through her attentiveness towards Pia’s story.

The dialogical approach can be said to go against common scientific practice in health care and medical research. Following in the steps of Frank and Nyheim Solbraekke (2023), we offer instead something that may be termed a performance, shedding light on a non-objectifiable subject position. We do not strive to find out something that is already there, awaiting discovery by the researcher. Instead, our research seeks to show how all of us as subjects participate in the elaboration of what is coming to be, a “subject [that] is both formulated by institutional and discursive conditions, and also continually formulating in her dialogical responses to responses” (Frank and Nyheim Solbraekke 2023, 80).

## Experiences of Vaccine Side Effects

In this section, we will present the dialogue to the reader divided into three themes: “The difficult story of lifelong suffering,” “Let’s wait and see,” and “We really didn’t mean to.” Each theme will be introduced by a short description.

## The Difficult Story of Lifelong Suffering

In this section of the dialogue, the reader will learn more about the grief and guilt that Pia has experienced during the last decade since Axel fell ill. It is a narrative that does not conform to the basic storylines in either the “hopeful quest narrative” or the “restitution narrative” of illness; rather, it is a chaos story with no promise of recovery (Frank 2013). However, this does not exclude feelings of hope, pride, commitment, and—needless to say—love.

MIA-MARIE (M): I don’t remember exactly, but someone said to me: “Yes, Pia has a son who got narcolepsy from the swine flu vaccine.” And I just froze for a second. And then I think I talked to you directly about it, at the first opportunity, that I said, “Maybe you and I can talk more about this, because my research is on vaccines and vaccine attitudes.” I remember that I felt a mix of eagerness to talk to you about these experiences your family has had, and also a wariness, because one doesn’t know how much people want to talk about illnesses in their families. But you were immediately so open, and said, “I’ve written a book about these experiences.” And it lies here between us now. […]

PIA (P): Yes. And I think I gave you the book pretty quickly after, too.

M: Yes, you did. Like, I got it right after.

P: Yeah, at our next meeting.

M: And before that meeting, I had also listened to your second book [*laughs*] […]. And I was impressed by your poetry; I thought it was touching and strong. And so, I got particularly curious about this book that is about the narcolepsy experiences in your family. I just want to say that I really appreciated it that you were so open. It felt like a great asset to me.

P: Yes? [*laughs*]

M: Yes. In fact, despite many interviews [with vaccine critics], I haven’t met anyone with children who suffer from narcolepsy before.

P: Yes, right. And I thought it was very exciting to meet someone who is researching this. […]

M: Before I met you, I maybe had a slight tendency to think, like many others, I guess—because this is what I hear when I talk to people about my research and incidentally mention narcolepsy—“Right, but there were so few children who were affected.” And we have recently looked at the numbers, and there are currently around 400–500 cases of narcolepsy as a Pandemrix-caused injury [in Sweden]. But getting to know you and your family—I’ve met your son, too—it has put that number into perspective. I can no longer see what happened as a small problem but instead as a very large and comprehensive problem. […] So I have gained perspective there because it is no small matter.

P: Mhm. When the COVID-19 pandemic came, I started processing even more what we have been through, and it came out as poems. […] And here is a poem I wrote about how big of a problem this is:

“Five hundred affected children,

living eighty years of illness,

make forty thousand years of suffering.

That is a

high price

for a society.”

M: Right. Most of those affected were children with a long life ahead of them.

P: Yes. And it’s not a fatal disease; it’s a brain injury that one has to live with. It doesn’t get better. It’s a question I sometimes get: “Axel is better now, right?” But no. It doesn’t go away. It is what it is. And this is the case for all these children. In a prosperous country like Sweden, people usually live long, and that entails many years of suffering from this illness. That is perhaps the most tragic in this, that it was [mainly] children who were affected. And that they have a long life ahead of them, living with the disability of this disease. And maybe not everyone thinks about that. Most of these children are young adults now. They will be middle-aged. They will grow old. And as they do, they will carry the narcolepsy and all the symptoms that come from it with them.

M: In everyday life, I also meet people who, when they hear about my research, say: “Yes, but they had a genetic mutation that caused them to get affected.” Again, I feel that people are looking for protective explanations that also ensure that they themselves or their children will not suffer similar vaccine side effects in the future. Do you get what I mean?

P: Mhm. That so-called genetic mutation is rather a tissue disposition that about 20% of the population has, so it’s not just a few people who have the disposition to be affected. But I understand what you mean, that there are many people who think “This won’t happen to us!” “This won’t happen to me!”

M: But it happened to you.

P: But it happened to us. So, it doesn’t make sense to think “It won’t happen to us.” It happened to us. And things happen to “us” too, they don’t just happen to others.

M: No, it happens to “us” too, yes.

P: And one never knows in life what is going to happen, of course.

M: No.

P: So, one can never say, “That won’t happen to me!” because we don’t actually know that.

M: We don’t know that.

P: […] And I think about it sometimes, that it took us a very long time to react. My son developed symptoms when he had just turned six, and it basically took us two years before we sought medical care for his symptoms. It was subtle in the beginning. He had stopped taking naps at daycare but started taking naps again, and then started falling asleep in any situation where it was a bit quiet—in the car, in shops, or at airports.

M: The way you described it, he could even fall asleep on some shelf, almost […] You took funny pictures of him when he was sleeping.

P: Exactly, we took funny pictures of him falling asleep in strange places. And he fell asleep so deeply that he was like a sack of potato flour when we tried to lift him. There was a different tonus in him. He felt different when we lifted him.

M: Didn’t you get worried then?

P: No, somehow not. I was also in the middle of a very busy period in my life, when I was doing an incredibly large number of projects, and I had started an interest association for cancer rehabilitation for various professions. There were so many things going on in my life. I was working more than full-time, with three kids, and…there wasn’t much time to think. And I realize in retrospect that our entire family life was structured so that there was no room for trouble.

M: Exactly. It was tight.

P: It was very tight! It worked as long as everyone was healthy, and our kids were almost always healthy, so we were always in motion, all of us. Our life was shaped after that. So, the idea that it could be something serious was very foreign to us. But, of course, I dealt with enormous feelings of guilt afterward. And it affected the whole family, the stress and worry for Axel made me ill and unable to work for more than a year. Since then, we also have had less time for his brothers.

And I’ve also thought that it was lucky that it wasn’t a brain tumor that we ignored for two years. But narcolepsy doesn’t change.

M: No, it doesn’t change.

P: No, it could not be influenced. Because the medicines are only for symptom relief […].

M: It’s a chronic disorder.

P: It’s a chronic disorder, yes, exactly. But on the other hand, it was sad for my son who couldn’t learn to read, because as soon as he sat down to practice his letters, he fell asleep. Always, every single time. And he is a very intelligent person.

M: Indeed, he even had a 2.0 on the SweSAT!^12^

P: He had a 2.0 on the SweSAT, for example! [*laughs*]

M: Which he managed to sit through, too! [*laughs*]

P: Yes, that was really the bigger challenge, staying awake.

M: But he laid down on some bench and slept for a while, and he took some extra medication?

P: He did take naps, but they were very short during this test compared to the naps he usually takes. And then he boosted with some extra medicine and managed to stay awake. And not just that, but he also managed to show, as we already knew, that he is very smart. And that’s why it was even stranger that he couldn’t learn to read when he was eight years old. But as soon as he got medicine, it took him three days and then he could read a book. As soon as he managed to stay awake, he could do it. But that guilt I have…I have struggled with it a lot. […] At home, I am the doctor. My husband is not a doctor and not in healthcare, so I am the medically responsible one in our house [*laughs*].

M: Right [*laughs*].

P: And I have worried ever since he was diagnosed. I have thought about how this will affect his whole life. There are so many things he won’t be able to do or do only to a limited extent. “What will it be like when he’s 18, when he’s 38, when he’s 58, what will his life be like?” Now, he studies part-time, 75% at high school. And I’ve been thinking that he might find it difficult to manage a family life, that he perhaps never will be able to start a family, because he’ll not have the energy for it. He may not be able to work, or he may only be able to work part-time. And we see that now, that he has very high grades. He could have had a career that he will not be able to have now. Then again, I’m not saying that life is all about pursuing a career, there are many people who don’t pursue a career and still live very meaningful lives anyway. But I just mean that he doesn’t have the opportunities to live the life he should have been able to live. I think about that a lot, of course.

## Let’s Wait and See

This part of the dialogue explores how the swine flu memories affected our willingness to take the COVID-19 vaccinations, respectively. Having experienced a severe side effect, either yourself or as a close relative, may affect further willingness to take a vaccination when a new pandemic strikes. This is a pressing issue that we need to be able to talk about.

M: You also took to the pen during this ongoing pandemic, when you wrote about how this [narcolepsy as a side effect] has become a problem for everyone […].

P: Yes, we are members of the Association for Narcolepsy, and what was discussed there—already early on, before the COVID-19 pandemic—was that several [people] expressed hesitations to get vaccinated. […] I’ve heard people say or seen people write, “Our family will never get a vaccine again.”

M: Right.

P: Never again in their lives. […] And how this has developed during the COVID-19 pandemic, I don’t know.

M: But it was a strong attitude then?

P: It was their dominant attitude then, yes: “We will never put our family through this again.”

M: The price was too high?

P: The price was way too high. And many have also refused to use the symptom-relieving medications that are available because they have somehow rejected all healthcare and all medications. “All medicine is only bad. It only exists because pharma companies want to make money.” And everything has side effects, of course. If you research any medicine, there’s a section about side effects. And these parents have developed a tendency to just look at “What are the side effects of this medicine?” and not consider what positive effects it brings. Our son has used the medicines that are available to have as good a life as possible. So, he can be awake during the day due to the fact that he sleeps more deeply, with the help of what you can call anesthetics, at night. And he can be awake and active for most of the day, even though he has to take naps, and even though he has a much lower energy level and can’t really use his full cognitive capacity because his short-term memory and working memory are affected by him being constantly tired. He has a hard time doing schoolwork the way he otherwise could have, but still, it works out somehow. […] But some people think, “We’re not going to use any medicine at all after this!” I know there are families who think that way. And this means that some of the children suffering from narcolepsy have not received the symptom-relieving medications. They have had to live their lives in hibernation, where they sleep for about 14–16 hours a day […].

M: Ugh.

P: Yes. And I can understand that, because all symptom-relieving medications that one can take are classified as narcotics. And it is natural that people are extra—

M: Worried?

P: Extra worried. […]

M: But it’s sad at the same time.

P: At the same time, it is very sad. Those children have had worse symptoms and [worse] lives than our child has had. Most importantly, the medicines have helped against the cataplexies in our son’s case. He no longer has cataplexies, and because of that, he is now able to take driving lessons to prepare for taking his driving test and obtaining a driver’s license.

M: As long as he takes medication, he can…

P: Yes. And I think of all the children who have not been getting help from medicines. And perhaps I, as a doctor, can differentiate more easily—for me, all medicines are not the same, nor is all healthcare the same.

M: No.

P: For me, they are very different things.

M: Right. You have benefited from being a doctor, in that way.

P: Yes, I have really benefited from that.

M: I can imagine that.

P: That I can distinguish between what happened with this vaccine […] and other medicine. For me, they are completely different things. And I also have enough insight into how the pharmaceutical industry works to be able to have some sort of a balanced view of…

M: A rather sober view.

P: A rather sober view, yes. Because, of course they want to make money, they are for-profit companies. But at the same time, we wouldn’t have had all the medicines we have today without their research and their… sure, it is profit-making but also very strictly regulated business, at least in Sweden. They can’t do things just to make money, either.

M: No, exactly.

P: And it’s part of my job as a clinically working doctor to take this into account. […]

M: While some others who have suffered these side effects put everything under one roof?

P: Yes, and perhaps think more in line with conspiracies.

M: Yes, right. You, as a doctor, are vaccinated against that, one might say [*laughs*]?

P: You can say that in a way, yes. Because I don’t think that everything is one big conspiracy; I rather think that we are all human, even those who work for pharma companies. They also want to do good; they want to do good things that help people. And they also want to make money.

M: It is complex.

P: It is complex. Yes, but that’s how I think. It has many sides […]. One of my poems that came to mind now is this one:

“Two parallel tracks inside me.

One track of reason

and one of emotions.

They refuse to intersect.

Reason says:

the only sensible thing

is to protect yourself.

Emotions say:

no.

They won’t listen to reason.”

M: And it was about the COVID-19 vaccine, that you wrote this?

P: Yes, it was about COVID-19. And that, I think, is so important to take into account when we talk about [vaccine hesitancy], when we think that the action needed is to inform these vaccine hesitant people better, and that they then will understand, when they have the facts. But it’s not just about that. It’s about this emotional side, too.

M: Absolutely, of course it is. And getting back to what we talked about. You have told me that your family is vaccinated against COVID-19.

P: Yes, that’s right.

M: Can you explain a little more?

P: Yes, for us, and for everyone else [affected] who had Pandemrix in mind, the new vaccines became a bit more complex. […] And then you should keep in mind that during these ten years that have passed, our children, including Axel, have continued to follow the national vaccination program for children. And they have all received travel vaccines. I’m an oncologist, so even before boys were recommended the HPV vaccination—when it was decided that only girls would get it—then I made sure to privately vaccinate them against HPV at our own expense. Because I thought, “They should get the same protection against cancer that all girls get.” So, I am in no way against vaccines. But of course…what became the tricky part was that it needed to happen so fast, producing the [COVID-19] vaccines. And I saw that as a risk.

M: That is the difference between pandemic vaccines and other vaccines.

P: That is the difference, because [with other vaccines] you have time to do all these tests and clinical studies on huge numbers of people, and so on. It had already been done as far as the HPV vaccines were concerned, for example. Whereas here [with COVID-19], for me it was probably more like, “How quickly do we dare to vaccinate ourselves?”

M: Mhm, right.

P: And I have a text about that, where I have written: ‘Those who have the most to gain may try first, and the rest of us can follow when the coast is clear.’

M: [*laughs*]

P: And that was the difference in my own personal strategy, in comparison with the swine flu vaccine. I thought, “Now they have developed these vaccines, and it is probably good that we have some different vaccine alternatives, and there are reports continuously coming in because there is such a strong focus on side effects now.” And I had a thought in the back of my mind, that enough time must pass, and enough people must get vaccinated, in order to detect such an unusual side effect. My own personal limit was that more than six months must have passed since one million people were vaccinated.

M: Mhm.

P: Because then, they would have discovered these unusual things that take some time to develop.

M: Neurological diseases?

P: Neurological diseases and such.

M: They have to show symptoms first.

P: They must have time to show symptoms, yes, exactly.

M: It took some time before we understood—

P: With narcolepsy?

M: —that narcolepsy was a side effect.

P: Yes. And then it was also our own experience—for our son, it took a couple of months before he actually showed the first symptoms […]. So, there are two different parts to this: first, that the symptoms do not develop immediately, it takes some time; and second, how quickly they come to the attention of the healthcare system. And it can take a long time, by all means. But for my own part, I thought that six months was enough if a very large number of people had been vaccinated.

M: Did you take all this into account?

P: I had all this in mind, yes.

M: So if we go back to the swine flu ten years earlier, then you didn’t reflect?

P: Then I didn’t think, no.

M: Not in that way at all?

P: I didn’t reflect in this way at all then. But now I did. And what happened in concrete terms was that I, as a healthcare professional, was offered to vaccinate against [COVID-19] already in February [2021], but then only three months had passed, so I did not want to vaccinate myself.

M: I see.

P: I thought it was too early.

M: Was it difficult to make that decision then?

P: Yes, it was perceived as quite strange, it was a bit controversial among my colleagues, that I can say.

M: But you could explain why?

P: Yes, I said that I was waiting because I am not in a risk group, so I want to wait for the public vaccination.

M: But everyone knew why?

P: Those I talked to about this knew why.

M: But maybe not everyone then?

P: I can say this: I was lying a bit low.

M: Okay!

P: I didn’t tell that many people. I told the head of my department, who was the one arranging [the vaccination], who asked “Who wants to get vaccinated?” Because they had to arrange this, and the logistics involved were like no other.

M: Yes, yes.

P: So he had to know how many people wanted the vaccine, and I said “No, I’m not interested in this vaccination round. I want to wait until the public vaccination.” Because it would start in May [2021].

M: And then a few months would have passed?

P: Then my six months would have passed as I wanted [*laughs*].

M: Yes, exactly [*laughs*].

P: So that’s actually what happened, that I delayed it for my own sake. And for our children, it wasn’t until the fall that they were offered vaccines.

M: It came late. Yes, exactly.

P: And then the Association for Narcolepsy had a webinar, because all these children naturally had many thoughts of their own about what to do. Our son, he was only 16, but he got to decide for himself. And then it was good that they organized this webinar where various experts in both infectious diseases and neurology discussed with the young people who had suffered narcolepsy from the Pandemrix vaccine, how to weigh the pros and cons, and what they thought were important aspects to take into consideration when making this decision. They collected all the facts available and presented them. And then our son made the decision: “I want to get vaccinated against COVID-19!” Because by then post-COVID had also become quite big.

M: Yes, yes.

P: And it turned out that, sure, the life-threatening disease, the life-threatening condition—that mostly affected the elderly, but quite young people could suffer from post-COVID.

M: Right.

P: Which has lots of different symptoms, including extreme fatigue. Which means very prolonged tiredness. And then our son said, “I already have extreme fatigue. I don’t want any more of it. Then my life would become completely impossible.”

M: And brain fog and other consequences.

P: Which could be consequences of getting post-COVID, yes. So, then he felt very keen to get vaccinated. Not as a specific risk group, but rather when it was time for the young people to get vaccinated, he also wanted to get vaccinated, based on that aspect. It was a very well-thought-through decision on his part.

M: Yes, it was. You were well-informed. He and the whole family were well-informed.

P: Yes, and the world, I would say. The scientific world was also well-informed by then. And then it felt like… although nothing is certain in life…

M: No, it isn’t. We take risks. … I myself never hesitated to be vaccinated and did not wait. I took the vaccine as soon as it was available.

P: It was in May, then. So it was around the same time that I took it.

M: It turned out that way, but I wasn’t thinking along those lines. I wasn’t thinking like a medical doctor, like you are [*laughs*].

P: No, exactly [*laughs*].

M: I just thought, “This is going to be fine!” and didn’t feel worried at all. I was just happy to get vaccinated. But when my then 12-year-old daughter was going to get vaccinated, when that offer came from the school, then we had a conversation about it at home. We had had COVID-19 and I was aware that one has strong antibodies then, on par with the antibodies one develops from the vaccine. So, we had a conversation about it at home. My husband thought it was a completely unnecessary conversation. “Why should we even talk about it? Of course, she will get the vaccine!” And I said, “But she already has antibodies. With that in mind, does she really need to get vaccinated, with the potential risks involved?” And then we had a discussion about risks. And I said, “I don’t know what the risks are. But we didn’t know that back then either, in 2010.” So, for me, it became a question. In the end, it was actually the 12-year-old herself who made the decision [*laughs*]. And she absolutely wanted to get vaccinated. And it went well. And then my son, when he turned 12, also got vaccinated. But it’s interesting, because there was still this thing with children and vaccines. For me, it was in the back of my head. Maybe I, as a parent, needed to think through this decision, as an extra precaution? And I had no such thoughts in 2010.

P: No.

M: In 2010, we vaccinated our very young children. At the time, my daughter was ten years younger, she was tiny then. But we made the decision to vaccinate her.

P: Mhm.

M: And I was pregnant during the swine flu with my third child, and it was said that pregnant women should absolutely take the jab, it was a special recommendation for us.

P: Yes, exactly. Yes…

M: So we all got vaccinated.

P: Yes, exactly. And now there was instead a discussion about whether you should vaccinate the children.

M: Yes, there was another kind of discussion about that.

## We Really Didn’t Mean to

The third and last theme explores Pia’s longing for official recognition of the Pandemrix tragedy: a wish for the politicians and civil servants to show themselves also as fellow human beings. It is not a matter of searching for scapegoats. Rather, it is—again—a longing for dialogue in the sense that the public employees have had many possibilities to confirm the suffering that not only the affected but also their families have endured, opportunities that they, to a high degree, have let pass, according to Pia.

P: What would have helped me as a mother, and what I think would have been important [for the authorities] to say, would be something like “It’s really incredibly tragic that this happened to these children and young people. And because that happened, we have…” The first thing is to acknowledge the emotional trauma. The second is, “Because this happened, we have learned very important lessons from it.” […] I don’t know what they were thinking, if they were thinking, “We’ll just never say it out loud. No one should be able to quote that we said anything about narcolepsy or Pandemrix or anything.”

M: Yes, it raises those kinds of questions.

P: Yes, it raises such questions for me. Why not seize the opportunity?

M: What did that strategy actually look like?

P: And then comes my question: Was it a conscious strategy? “Don’t poke the bear!” Or was it an unconscious strategy that is also rooted in a trauma? Because I think it has been traumatic for those who planned the whole swine flu vaccination. It was traumatic for them, too, that this affected children. They must also have felt guilty, or struggled with what it was they were feeling, and how guilty they should feel, and, I figure, tried to push it away, “Yes, but we couldn’t know that, and we did our best,” and so on. But this is a wound for them too. That’s how I think. And I have a poem about that too, we’ll see if I can find it…

M: I know which one you mean.

P:

“Deciding for all the country

cannot have been easy.

All those wanting

to do good work,

who found themselves to be

civil disservants.

I believe their nights

are anguished too.”

M: Mhm.

P: Another related poem is:

“It was such a good plan,

such resolute acting.

We all stood united,

contributing to a good cause:

protecting each other,

protecting the children.

Our intentions were the best,

still, it went wrong.

Whatever shall we do

the next time?”

M: And that’s where we ended up, ten years later.

P: And then we ended up here. […] And yet we don’t address this wound. We don’t touch it. It could have been an opportunity for healing, actually. If they would also show that “Yes, we mourn this. We also want to help, of course, much more than what has been done so far for those who were affected.” One more poem:

“The dormant children,

no longer able to laugh.

They didn’t get to choose.

We chose for them,

and we now stand guilty.

That guilt is crushing us.

We didn’t mean to!

We really didn’t mean to.”

P: What [Minister of Social Affairs] Göran Hägglund did at least, was that he said, as an official, “We admit that this is a vaccine injury that has affected all these children. We officially confirm it,” so to speak. And then one could get some compensation.^13^

M: Mhm.

P: But there is still this inability to touch it, the inability to put it into words. The inability to grieve, together, what has happened, which I think is one of the big problems with this Pandemrix question. Because, if we had been able to process it emotionally together by putting it into words and facing the sadness it evokes, then we might have been able to heal. Instead, now it’s this psychological trauma that cannot be touched, cannot be processed. It’s like little capsules affecting everything around them, that are invisible and impossible to integrate.

M: Like sealed capsules that still leak, right?

P: Yes, or there’s rather this pressure wave around them. It’s like a foreign body in a tissue that causes inflammation and infection around it, so to speak. I see this as a trauma in the sense that it is an event, a foreign body, which comes into our lives and causes a great number of consequences around it, precisely because it cannot be processed, because it just lies there. And especially, it cannot be integrated into one’s life and history. What is characteristic of it is that it cannot be talked about. It cannot be touched; it cannot be thought of. People spend a lot of time pushing it away, the memory of it.

M: Exactly.

P: And as your research shows, some are using this event as a reminder when talking about the COVID-19 vaccine. But they are not processing the trauma. They don’t talk about what narcolepsy means or how to live with it, and how sorry we are that it happened, and so on. It’s more like, again, a kind of…there are flashes of it, we briefly bring it up, but then we push it away again. In psychological terms, you can put them in opposition to each other—things that can be mourned and integrated, versus things that happen and just arouse, like, anger.

M: Or discomfort.

P: And discomfort. And that we push away. It’s like two different ways of dealing with things.

M: Getting back to something you said. Because you said that one gets hurt, when, for example, a civil servant or a politician […] doesn’t use emotional language. Why is it important?

P: It’s about getting recognition from people who have been involved in what has happened. To have an acknowledgment of how tragic it actually is that this happened. How sad it is. And when one switches those feelings off and just sticks to political strategies, communication strategies...

M: Right. Regardless of the reason.

P: I could try to understand why they do it. But it results in feeling…our suffering…it feels like it’s being ignored, so to say.

M: You need affirmation for your suffering.

P: It would have been helpful for us in some way, I think, if someone had said: “This was terribly tragic, what happened, but we have learned so many important lessons from it, and we have now acted differently.” Then one could have thought that yes, it is still just as tragic that it happened, but at least it made a difference. […] But now it’s like “No, we don’t want to think about what happened. We don’t want to think about you who are affected. We just want to bury it all and forget about it.”

M: And then the difficult question, which I think my informants [vaccine critics] would have asked in relation to this, is: “Does it help us who are already skeptical of vaccines that you are so silent about such an event? And if such a strategy has been chosen, is it the right strategy?” Just like you say, I can fully understand how they might think that they don’t want to spark more vaccine criticism than we already have to deal with. They do not want to reinforce the skepticism that already exists against vaccines.

P: I can understand if they don’t spontaneously bring it up at press conferences themselves. But if it has already been mentioned by the media, […] then it is about the officials asking themselves the question: “How should I deal with the fact that we are already talking about narcolepsy?”

M: Yes.

P: And in that situation, not daring to address it in any way—that’s downright counterproductive and destructive, I would say, even if one may have adopted a “don’t talk about it” strategy. […] If [the media] is already talking about it, if you as an official cannot avoid the topic—if the topic of conversation *is* narcolepsy—then you could use a completely different strategy. And that’s what makes me think that maybe they’re still so emotionally blocked and so affected by this that it isn’t possible [to talk about it]. I don’t know. Or there was just this strategy, and they stuck to it, and then it comes out totally wrong, completely insensitive. It appears to be completely insensitive. […] Also, a common term for it would be helpful. Could they have, for example, called it the Pandemrix tragedy, or the Pandemrix catastrophe, or the Pandemrix something? That would have shown that…as with Thalidomide, we are talking about the Thalidomide scandal [Neurosedynskandalen in Swedish, where pregnant women were recommended to use Thalidomide, which disfigured the children they carried].^14^ In Sweden, Pandemrix affected five times the number of children who were born with malformations due to Thalidomide. For me, Pandemrix *is* a catastrophe of sorts. I believe that such a designation would have strengthened the confidence in the authorities’ actions.

M: You touch on something interesting here that I have reflected a lot on in light of this conversation and other conversations we’ve had—the fact that civil servants are both civil servants and people. Right?

P: Yes.

M: It goes without saying that they have also been affected by this. Of course, it has affected them. But only one state official, Göran Stiernstedt [coordinator of the mass vaccination] has said it outright.^15^

P: He talks about feeling guilty. […]

M: He involves himself in that interview.

P: Yes, he does.

M: He is actually emotional. Even if it’s somewhat between the lines, he still puts it into words.

P: No, it’s not between the lines. He says straight out that he feels guilty, and that he has taken it very much to heart.

M: Yes.

P: And that has been very important to me at least. That emotional and moral recognition.

## Discussion

In this article, through a dialogue, we have highlighted the cultural and social aspects of severe vaccine side effects by exploring what a disease like narcolepsy does to someone’s life, how intimate experiences of negative events following vaccination affect one’s willingness to take future vaccines, and how a tragedy like narcolepsy as a vaccine side effect can be collectively processed and remembered. With inspiration from Frank and Nyheim Solbraekke (2023), we have offered a performance that sheds light on a non-objectifiable subject position, thus uncovering something not already present by employing a method that allows for the movement of thought.

Our study offers a complement to Lundgren’s work (Lundgren 2015a, 2015b, 2016, 2017) on Pandemrix-caused narcolepsy by providing significantly more space for subjective experiences. It should also be regarded as an extension of Lundgren’s research by connecting the experiences from the swine flu mass vaccination with the COVID-19 mass vaccination. We have shown how pandemic memories are formed collectively; events happening ten years ago are remembered and processed by people when standing before the decision once again—to take the shot or not. Concerning the COVID-19 pandemic, we are both convinced of the importance and effectiveness of mass vaccination to stop the spread of the virus, which until the time of writing this paper, has caused nearly seven million deaths around the globe (Johns Hopkins University & Medicine Coronavirus Resource Center 2023).^16^ And, to repeat ourselves, as long as serious side effects are not ignored, they can nurture reflexive awareness, empathy, and responsibility, which can strengthen public trust regarding possible future pandemic interventions. In line with Lundgren, we conclude that however disturbing it may be to recognize and take part in negative vaccination experiences, like the ones that Pia has testified about, they still have to be communicated openly and transparently.

In the first part of the dialogue, it becomes clear that narcolepsy brings a lot of suffering, not only to the individual but to the whole family. Even though the number of affected children is rather small, the years of suffering ahead of them and their close relatives cannot be thought of as a “small” problem. This theme also contains feelings of guilt; as a doctor, Pia had the medical responsibility for her family, or so she felt, a confidence that she, in retrospect, did not live up to according to herself.

The second section sheds light on the COVID-19 pandemic and how experiences from the swine flu mass vaccination affected the willingness to take the COVID-19 vaccination. Among other things, the reader learned that the Association for Narcolepsy, at the time of the mass vaccinations in 2021, arranged internal online seminars with invited vaccine experts to inform the affected children and their families. Pia also described how she cautiously managed the COVID-19 vaccinations that early on were offered to her at her workplace; the cultural and social expectations formed a pressure that she had to maneuver in everyday life.

The last part of the dialogue expresses, on the one hand, a need and a wish for recognition. Pia would like politicians and other officials to (1) openly address the narcolepsy problem; (2) recognize the tragedy and suffering of those affected; (3) explain publicly what they have learned from the Pandemrix catastrophic consequences; and (4) name the event so that it becomes real and memorable and, thus, healable.

On the other hand, Pia also evaluates public employees’ way of acting and talking in relation to one another, and we chose to keep their names in the manuscript. The claims that are being made are supported by links to newspaper articles. Still, someone else affected by Pandemrix in one way or another might, of course, have differing views on both the matter of recognition and how politicians and civil servants should have acted during the crisis and afterward.

Pia speaks as a subject, entitled to her own voice, and what the reader might not detect by reading—and not hearing—the dialogue is that her tone of voice was more encouraging than dismissive—but also hurt. More than anything, it contained frustration, pain, and sorrow caused by a feeling of standing alone with a life-long struggle for her son’s wellbeing and for his and the family’s right to public recognition and reasonable economic compensation. Mia-Marie participates in a dual role: as the active listener in the dialogue, curious and attentive towards Pia’s story, but also as a scholar in the field of vaccine hesitancy, with vast experience and understanding when it comes to conversations about these complicated issues.

Side effects are complex matters, spanning medical, biological, psychological, political, economic, historical, social, and cultural dimensions. To enhance vaccine confidence, all of these dimensions need to be explored. We have contributed to new knowledge on several of those dimensions. It is our belief that conversations on side effects led by so-called anti-vaxxers need to be counterbalanced and that medical humanities scholars, reaching across academic disciplines and between academia and society, have a role to play in this regard. This has been our contribution.

## Data Availability

The dialogue will be archived at the Folklife Archives at Lund University.
